# Direct Measurement of Aerosol Liquid Water Content: A Case Study in Summer in Nanjing, China

**DOI:** 10.3390/toxics12030164

**Published:** 2024-02-20

**Authors:** Daoming Li, Shijie Cui, Yun Wu, Junfeng Wang, Xinlei Ge

**Affiliations:** Jiangsu Key Laboratory of Atmospheric Environment Monitoring and Pollution Control (AEMPC), Collaborative Innovation Center of Atmospheric Environment and Equipment Technology (CIC-AEET), School of Environmental Science and Engineering, Nanjing University of Information Science and Technology, Nanjing 210044, China

**Keywords:** aerosol liquid water content, visibility, ambient PM_2.5_

## Abstract

Aerosol liquid water content (ALWC) affects the mass loading, optical properties, and toxicity of aerosols. However, the measurement of ALWC is very rare due to its requirement of sophisticated instruments and its high operational costs. In this work, we improved on our previous simple, low-cost method by using a combination of one real-time fine particulate matter (PM_2.5_) monitor and two turbidimeters and successfully applied these for the direct measurement of ALWC in PM_2.5_ in Nanjing during the summer of 2023. The average ALWC during this measurement period occupied ~1/6 of the total PM_2.5_ mass, and this contribution was even greater with the elevation in the PM_2.5_ concentration. The ALWC was, as anticipated, closely related to the relative humidity (RH) and PM_2.5_ concentrations, but it did not always increase with the air quality index (AQI) due to the fact that polluted periods in summer were often governed by high O_3_ levels, not PM_2.5_ levels. The ALWC also had a great impact on visibility; it could decrease the visibility rapidly to hazy conditions when the dry PM_2.5_ was not high (~30 μg m^−3^) or the AQI was “good” (75~100), indicating that the air quality classified as “good” using the dry PM_2.5_ concentration might actually be “lightly polluted” if the ALWC is included. We also found that the air mass originating from Northeast China had the lowest PM_2.5_ mass concentration yet the highest ALWC values due to its high RH. Moreover, the quantification of ALWC levels can help us understand the solubility/bioavailability and thus the toxic effects of some specific components (for example, heavy metals or organics). Moreover, the influence of ALWC on air quality classifications should also be considered in the assessment of the health effects of air pollution and in public health early warning and protection.

## 1. Introduction

Water is a ubiquitous component of fine particulate matter (PM_2.5_), referred to as aerosol liquid water (ALW). Water is also known to be a very unique species that can exist in gas, liquid, or solid phases in the atmosphere, and the ALW content (ALWC) is governed by the gas–particle equilibrium (GPP). In detail, the ALWC can be affected by the relative humidity (RH), air temperature, concentration, and chemical composition of PM_2.5_ as well as its phase state, etc. [1]. The ALWC can significantly alter ambient aerosol mass concentrations, particularly at a high RH [2,3], and plays a critical role in many atmospheric physicochemical processes [4]. It can impact the optical properties of aerosols, leading to increased light extinction, reduced atmospheric visibility, elevated aerosol optical depth (AOD), and variations in the direct impact of aerosols on the climate [5,6,7,8,9]. ALWC can also act as a site for multiphase/aqueous/interfacial reactions, perturbing particle/droplet chemistry and further affecting the aging process of aerosols [10], with many studies showing the importance of ALWC in the formation of secondary aerosol species [11,12,13].

Moreover, ALWC can also affect an aerosol’s solubility, which in turn influences the adsorption and dissolution of toxic components like heavy metal ions, etc. Solubility is an important indicator to link metals with their adverse health effects in epidemiological and toxicological studies [14]. Reactive oxygen species (ROS) produced by transition metal ions (such as cadmium and nickel) may trigger a wide range of health consequences including inflammation, DNA damage, cardiovascular disease, and acute heart disease [15,16]. Some other non-transition metals can be quite harmful to human health too; for instance, lead and arsenic are found to be highly carcinogenic [16]. Previous studies show that soluble fractions of Fe, Cd, Pb, and Zn increased significantly under acidic solvents, suggesting that acidic aerosols with an abundant ALWC are able to dissolve more trace metals [17] and therefore make the particles more toxic and pose a greater threat to human health than dry aerosols.

However, despite its critical importance, ALWC is often not routinely measured or included in reported PM_2.5_ concentrations. Most instruments determine the (dry) aerosol mass and composition after the removal of associated aerosol water. The ALWC is typically estimated based on the measured aerosol composition and concentration by using various thermodynamic models (for examples, the extended aerosol inorganic model (E-AIM) [18] and ISORROPIA II [19]). Such a methodology assumes a thermodynamic equilibrium state of the aerosols and often does not include all aerosol components (especially organics) [20,21]. A humidified tandem differential mobility analyzer (H-TDMA) can be used to determine the hygroscopic growth factor (HGF) of ambient aerosols under a fixed RH (typically at 90%) [22]; it can obtain the particle number size distributions [23,24,25] under both dry and humid conditions and then calculate the ALWC by comparing the volume changes before and after humidification [26,27,28]. The cloud condensation nuclei counter (CCNC) and electrodynamic balance (EDB) or optical tweezers can also be used to study the hygroscopicity (though they often are not direct measurements of ALWC) of particles [29,30,31]. In addition, sophisticated optical instruments, such as polarized lidar [32] or a three-wavelength humidified turbidimeter, use measured aerosol light-scattering coefficients and backscattering coefficients to estimate the ALWC [33]. It should be noted that the methods above are often complicated and expensive, and some of them are not applicable for the measurement of real air, therefore limiting their widespread use.

In this regard, our group previously established a novel, simple, and low-cost method [34] that showed that the “bias” in the measured PM_2.5_ mass concentration caused by a high RH in a turbidimeter from that measured at dry air conditions can be used to determine the ALWC. In this work, we further improved this method and for the first time, applied it to directly quantify the ALWC in ambient PM_2.5_ in Nanjing during the summer of 2023. Our findings are also insightful for our understanding of ambient aerosol chemistry and toxicology in similar large cities like Nanjing with relatively significant air pollution issues [35,36,37].

## 2. Sampling Site and Instrumentation

**Figure 1:**
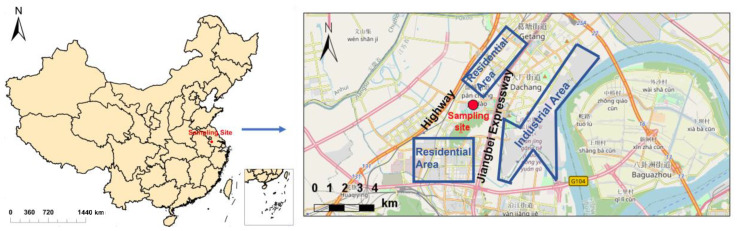
Location and surroundings of the sampling site.

The field campaign was conducted inside the campus of Nanjing University of Information Science and Technology (NUIST) (32°12′20.82″ N, 118°42′25.46″ E), located in Nanjing, Jiangsu Province, China (Figure 1). The measurement period was from 30 July 2023 to 1 September 2023 (34 days). Two identical aerosol monitors (Thermo Scientific™ (Waltham, MA, USA) Personal DataRAM^TM^ pDR-1500) (a type of nephelometric monitor, referred to as PDR hereafter) and one Thermo Scientific™ (Waltham, MA, USA) Model 5030i SHARP (Synchronized Hybrid Ambient Real-time Particulate Monitor, referred to as 5030i hereafter) were used. The sampling site is located west of an expressway (Jiangbei Expressway) and an industrial area (including Nanjing Iron & Steel United Co., Ltd. (Nanjing, China), Yangtze Petrochemical Co., Ltd. (Nanjing, China), and a number of other chemical and petrochemical engineering companies as well as power plants). The site is also surrounded by a residential area, and it is representative of the typical suburban environment with intense anthropogenic emissions.

The 5030i monitor combines the light-scattering nephelometry and beta attenuation technique and provides accurate, fast, and real-time PM_2.5_ mass concentrations with low detection limits. It reports the dried PM_2.5_ mass concentration, denoted as 5030i_PM. The two PDRs were placed parallel to one another and share a sampling line with a PM_2.5_ cyclone placed about 10 m above the ground. The sample air was dried (typically to an RH under 20%) and fed into a PDR, which measured the PM_2.5_ mass concentration, denoted as PDR_dry. We used a dryer filled with silica gel to remove the moisture from the air before entering into the PDR_dry. In order to keep the dryer operating effectively, we changed the silica gel at least every 2 days (sometimes more frequently according to the weather conditions). Another PDR was directly connected with the ambient air flow without the removal of moisture. Thus, the determined PM_2.5_ mass concentration was denoted as PDR_wet_original, and this PDR also recorded the temperature and RH of the ambient air. For consistency, the time resolution was set to 1 min for both the PDRs and 5030i. Other instrumental details about the PDR can be found in previous works [34,38,39].

The concentrations of common gaseous pollutants (NO_2_, SO_2_, O_3_, CO) were acquired simultaneously using a series of Thermo Scientific monitors. Meteorological parameters such as the wind speed (WS) and wind direction (WD) were obtained from an automated weather station (Vaisala WXT520) located at the same sampling site. Visibility data were obtained from a nearby national environmental monitoring station. Note all the data reported here are shown in Beijing time (UTC + 8).

## 3. Results and Discussion

### 3.1. Improvement of ALWC Measurement

**Figure 2:**
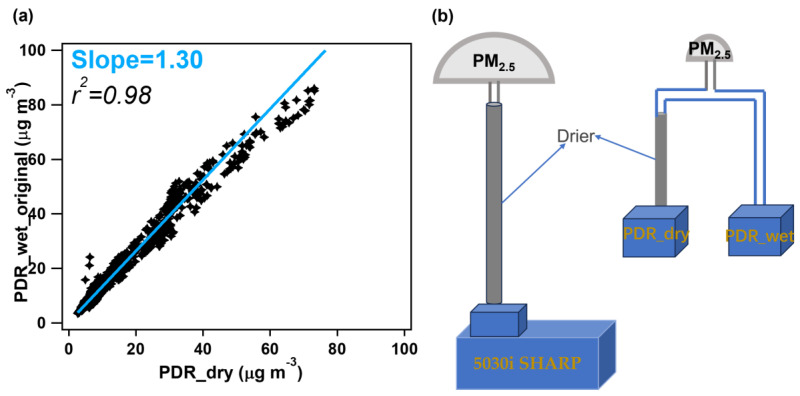
(**a**) Correlation of the PM_2.5_ mass concentrations (PDR_wet_original vs. PDR_dry) between the two PDRs at RH < 50%. (**b**) Simple schematic of the ALWC measurement method.

**Figure 3:**
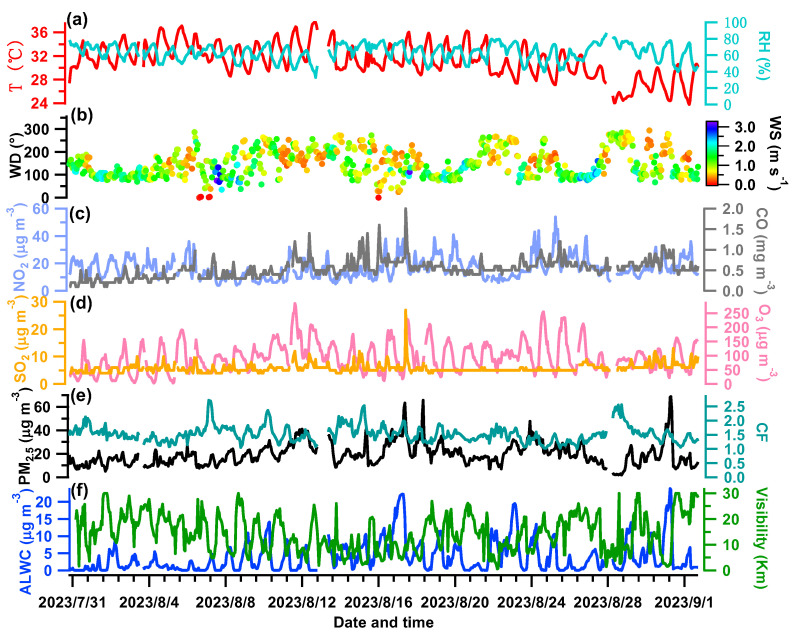
(**a**) Relative humidity (RH) and air temperature (T); (**b**) wind speed (WS) and wind direction (WD); mass concentrations of gaseous pollutants of NO_2_, CO (**c**), SO_2_ and O_3_ (**d**); (**e**) PM_2.5_ mass concentration measured by the 5030i and the calibration factor (CF = PDR_dry/5030i_PM); (**f**) derived ALWC values and measured visibility.

First, to ensure the consistency of the acquired data between the two PDRs, we compared the measured PM_2.5_ concentrations at an RH < 50% for the two PDRs, as shown in Figure 2. The correlation between them is very tight, with a Pearson’s r^2^ of 0.98; the slope of the linear fit is 1.3, denoted as *c* in Equation (1). This value represents the instrumental deviation of these two monitors and is therefore used as a calibration factor to derive the real ambient wet PM_2.5_ mass concentration (PDR_wet), as shown in Equation (2). The reason that we used data at an RH < 50% rather than a smaller RH threshold (for example, 20%) is due to the fact that the RH during the measurement period was mostly higher than 40% (as shown in Figure 3a). Moreover, the regression of the very few data with an RH < 40% yielded a slope of 1.28 and r^2^ of 0.96, which are almost similar to those obtained under an RH < 50%; therefore, we used the calibration factor obtained at an RH < 50% for consistency with our previous work [34] as well as for a broader coverage of the measurement data and thus robustness of the regression. Note that this systematic difference is instrument-dependent; we thus recommend that users perform this calibration for each pair of PDRs that are used in users’ specific ALWC measurements. If there are enough data, we also recommend obtaining the calibration factor by comparing data at a smaller RH (such as 20% or 30%) to reduce the influence of possible absorbed water on the PDR_wet_original.

(1) $$c=\frac{PDR\_wet\_original}{PDR\_dry}$$

(2) $$PDR\_wet=\frac{PDR\_wet\_original}{c}$$

In our previous work [34], we used an Aerodyne high-resolution time-of-flight aerosol mass spectrometer (AMS) as a reference instrument to correct the readings of the PDR_dry to reflect the true fine PM concentrations. However, in practical terms, an AMS is a highly expensive and sophisticated instrument, requiring well-trained personnel to operate it [40,41], and it is not an instrument conventionally employed for atmospheric environmental monitoring. Moreover, an AMS typically measures submicro-meter particles (30~1200 nm) rather than PM_2.5_ due to the transmission efficiency of its inlet system [42], unless the PM_2.5_ lens system is adopted [43,44]. Therefore, instead of an AMS, we used the 5030i, which is broadly equipped as the routine PM_2.5_ mass monitor worldwide, as the reference instrument in this work. Similarly, we introduced the calibration factor (CF) (calculated as CF = PDR_dry/5030i_PM) to correct the PDR_dry and then to calculate the PM_2.5_ mass concentration under real ambient conditions (PDR_wet_ct) using Equation (3).

(3) $$PDR\_{wet}_{ct}=\frac{PDR\_wet}{CF}$$

Next, the ALWC is simply the difference between the PDR_wet_ct and 5030i_PM, as shown in Equation (4). The flow chart of the improved ALWC measurement method is shown in Figure 2b.

(4) $$ALWC=PDR_{\_wet_{ct}}-5030i\_PM$$

### 3.2. ALWC Results and Correlations with Other Variables

**Figure 4:**
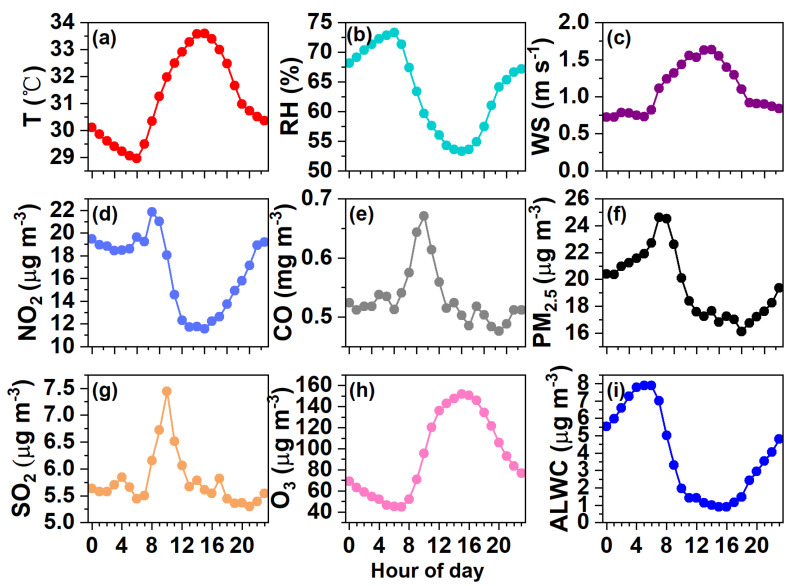
Diurnal variations in (**a**) Temperature, (**b**) RH, (**c**) WS, and concentrations of (**d**) NO_2_, (**e**) CO, (**f**) PM_2.5_, (**g**) SO_2_, (**h**) O_3_, and (**i**) ALWC.

Figure 3 shows the meteorological parameters, concentrations of gaseous pollutants, dry PM_2.5_ (from 5030i), and ALWC over the measurement period. The averaged diurnal patterns of relevant variables are shown in Figure 4. The campaign-averaged temperature was 31.19 °C, and the mean RH was 63.42%, indicating hot and mildly humid weather overall. The temperature peaked in the afternoon while the RH was relatively high at night (Figure 4a,b). The wind was often from the southeast (with an average of 148.6°). Its speed was low (an average of 1.11 m s^−1^ and a maximum of 3.22 m s^−1^) and it was particularly weak at night (~0.75 m s^−1^), coinciding with a high RH. This indicates that stagnant and humid air conditions therefore were unfavorable for pollutant dispersion.

The SO_2_, NO_2_, O_3_, and CO concentrations were averaged to be 5.76, 16.64, 94.56 μg m^−3^, and 0.53 mg m^−3^, respectively. The daily variations in SO_2_ and CO are quite similar (Figure 4g,e), likely indicating their similar sources from the nearby industrial area. The daily NO_2_ pattern was a bit different as it remained high after midnight (Figure 4d). The daily trends of O_3_ were opposite to those of NO_2_ (Figure 4h), reflecting the consumption of NO_2_ to produce O_3_ [45]. The average PM_2.5_ mass loading was 19.50 μg m^−3^, ranging from 1.87 μg m^−3^ to 68.65 μg m^−3^. On a daily basis (Figure 4f), the PM_2.5_ concentration peaked in the morning (24.60 μg m^−3^ at ~7:00 am) and reached a minimum of 16.12 μg m^−3^ at ~18:00 pm, partially owing to influences from the WS, RH, and variations in planetary boundary layer height.

The determined ALWC varied from 0.003 to 23.84 μg m^−3^, with an average of 3.85 μg m^−3^. The ALWC occupied on average 16.5% of the total aerosol mass (23.35 μg m^−3^). The visibility varied from 0.51 to 30 km, with a campaign mean of 14.37 km. The temporal trends of the ALWC and visibility are the opposite of one another as illustrated in Figure 3f, demonstrating the significant role of ALWC in visibility degradation (see our further discussion in Section 3.4). The diurnal trend of the ALWC (Figure 4i) naturally resembled that of the RH (Figure 4b); it peaked at ~6:00 am (7.88 μg m^−3^), reached the lowest value at 15:00 pm (0.90 μg m^−3^), and then began to rise in line with the increase in RH and decrease in temperature (Figure 4a,b), which favored the partitioning/condensation of water vapor onto particles [23,26].

**Figure 5:**
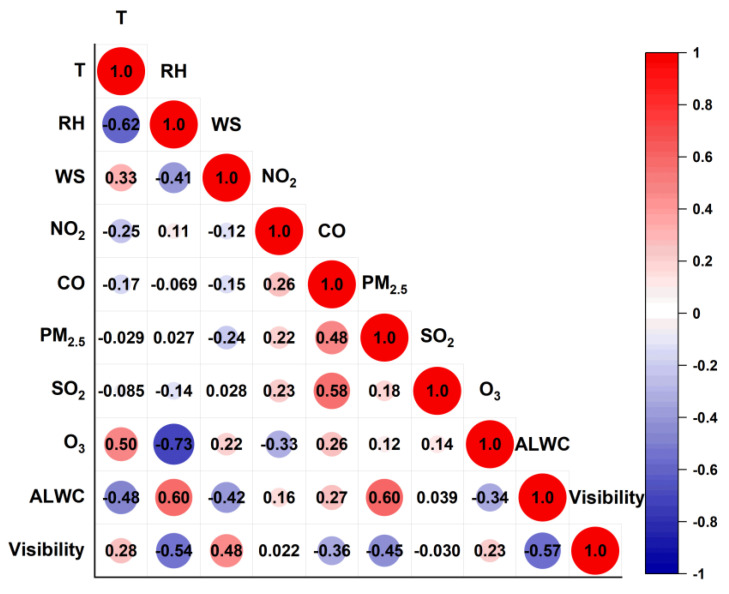
Cross-correlation coefficients (Pearson’s r) among gaseous pollutants (NO_2_, CO, SO_2_, O_3_), RH, T, WS, ALWC, and visibility.

Figure 5 further presents the cross-correlation coefficients among the temporal variations in the measured variables. The ALWC correlated positively with the RH (r of 0.60) and PM_2.5_ (0.60) as expected, but negatively with the WS (−0.42) and T (−0.48) as well as O_3_ (−0.34). The results show clearly that the PM_2.5_ concentration and RH govern the ALWC, while high temperatures and winds accompanied by high O_3_ values on the contrary promoted the evaporation of the ALWC into the gas phase. The visibility mainly showed positive correlations with the WS (r of 0.48) and T (0.28), indicating that the visibility is great in hot weather with high wind speeds. However, the visibility was negatively correlated with the ALWC (r of −0.57), RH (−0.54), and PM_2.5_ (−0.45), indicating heavy impacts from the ALWC and PM_2.5_ loadings.

In Table 1, we compare a few key studies regarding the ALWC in the Yangtze River Delta (YRD) region. Note that all these ALWC values are estimated from thermodynamic models. Xian et al. [20] calculated an average ALWC of 5.33 μg m^−3^ in Nanjing during the summer of 2021. Considering the higher RH (69.05% vs. 63.42%) and similar PM concentration (18.88 vs. 19.50 μg m^−3^) in their study, a higher ALWC is reasonable and vice versa. Their work validates the measured values in this study. The ALWC levels in Shanghai, Jiaxing, and Huangshan areas were all higher than the values in this work, due to both higher PM_2.5_ concentrations and higher RH values. Of course, other meteorological factors and more importantly aerosol compositions can affect the ALWC too [4].

### 3.3. Characteristics of ALWC under Different Pollution Scenarios

**Figure 6:**
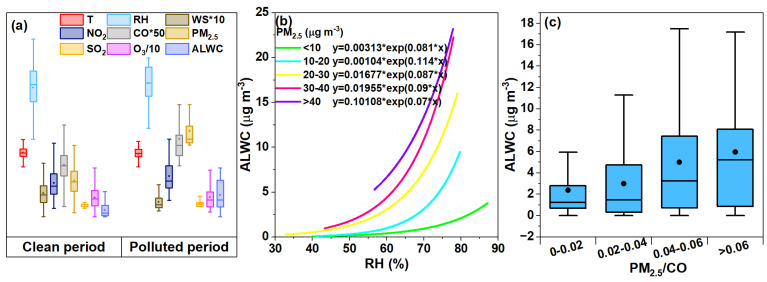
(**a**) Distributions of ALWC, PM_2.5_, gaseous pollutants (O_3_, CO, NO_2_, SO_2_), and the key meteorological parameters (WS, RH, T) during the clean and polluted periods, respectively. (**b**) Exponentially fitted equations (y = a*e^b*x^) of ALWC versus RH under different PM_2.5_ levels (marked in different colors). (**c**) Variation in water content over a range of PM_2.5_/CO ratios.

According to the current China ambient air quality standard (CAAQS), we classified the sampled period into “clean” (24 h averaged PM_2.5_ < 35 μg m^−3^) and “polluted” periods (24 h averaged PM_2.5_ ≥ 35 μg m^−3^). Figure 6a displays the distribution of the PM_2.5_, ALWC, O_3_, CO, NO_2_, and SO_2_ concentrations as well as the key meteorological parameters under the two scenarios. The meteorological conditions were more stagnant during the polluted period than the clean period, with a lower mean WS (0.73 vs. 1.14 m s^−1^), higher mean RH (65.65% vs. 63.24%), and lower mean T (30.83 vs. 31.22 °C). The four gaseous pollutants increased in varying degrees from the clean to the polluted periods too. The average PM_2.5_ concentration was 17.79 μg m^−3^ during the clean period and increased to 41.95 μg m^−3^ during the polluted period, which was lower than the PM_2.5_ levels in the summer of 2014 in Nanjing, indicating an improvement in air quality [47]. Correspondingly, the ALWC during the polluted period was 3.15 fold that of during the clean period (10.55 vs. 3.35 μg m^−3^); the mass fraction of the ALWC to the total PM_2.5_ mass also increased to 20.1% from 15.85%.

Furthermore, we investigated the relationships between the ALWC and RH under different PM_2.5_ levels, which show non-linear exponential correlations as seen in Figure 6b. At low RH levels, the ALWC increased slowly with the RH, but much rapidly in regions with a high RH. This is likely due to the fact that the key hygroscopic aerosol components like nitrate and sulfate do not uptake water at a RH that is lower than their deliquescence points, but can uptake water exponentially when the RH exceeds the deliquescent RHs [23,25,26], though no clear threshold RHs can be directly observed in Figure 6b since ambient aerosols are a complex mixture of hygroscopic salts and a wide range of organics, etc. (many of them have no definite deliquescent RH values). The increase in ALWC become more rapid with the elevation in the PM_2.5_ levels, likely due to a two-way coupling effect between the ALWC and PM_2.5_, as an increased ALWC may promote aqueous/heterogeneous oxidation and the gas-to-particle condensation of some species, therefore increasing the PM_2.5_ mass, which in turn leads to more water uptake and further increases the ALWC [49,50,51,52]. Moreover, since large amounts of ALWC can increase the dissolution of metal ions or other toxic and hazardous substances, this result indicates that the same toxic species might be released more effectively in polluted than in clean air. CO can be considered as a tracer of the primary combustion source, and the ratio of PM_2.5_/CO can be used to qualitatively infer the contribution of secondary aerosols [53,54]. The mean value of PM_2.5_/CO in this experiment was 0.04 (0.004~0.190) which is consistent with that of a previous study [55]. From Figure 6c, it can be seen that the content of ALWC in general increased with the increase in the PM_2.5_/CO ratio (2.36~5.95 μg m^−3^), suggesting the possible presence of more accumulation-mode particles and secondary species during the polluted period, which indeed enhanced the water uptake of the aerosols [56,57].

**Figure 7:**
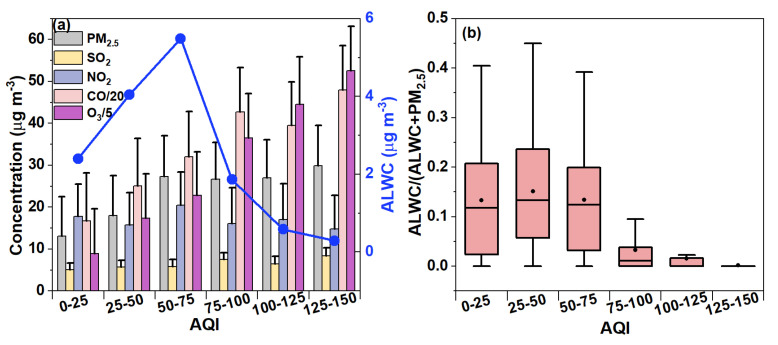
(**a**) Average concentrations of different gaseous pollutants (CO, NO_2_, SO_2_, O_3_) as well as PM_2.5_ and ALWC at different AQI ranges. (**b**) Distribution of mass fractions of ALWC to total PM_2.5_ at different AQI ranges.

The air quality index (AQI) is a combined indicator considering both particular matter and the major gaseous pollutants [58,59]. The mean AQI was on average 42.47, ranging from 12 to 142 throughout the measurement period, and only 3.57% of the AQI values were classified as “polluted”. Also, it should be noted that the AQI is mainly governed by O_3_ levels, owing to strong photochemical reactions between volatile organic compounds and nitrogen oxides during the summer in Nanjing, consistent with a previous analysis during the summers of 2019 and 2020 in the YRD region [60]. Here, we further explored the features of the ALWC under different AQI levels. First, the average concentrations of PM_2.5_, gaseous pollutants, and the ALWC at different AQI categories are illustrated in Figure 7a. The O_3_ concentrations continuously increased with the AQI, while the PM_2.5_ increased at an AQI < 75, but remained generally stable at an AQI > 75, showing that relatively large AQI values (more polluted air) were mainly affected by O_3_ not PM_2.5_ in this work. The behavior of the ALWC against the AQI resembled that of PM_2.5_ at an AQI < 75 (maximum of 5.49 μg m^−3^ for an AQI of 50–75), but decreased quickly and significantly at an AQI > 75 (minimum of 0.30 μg m^−3^ for an AQI of 125–150). This can be expected as high AQI values were associated with high O_3_ levels accompanied by high temperatures, strong winds, and low RHs (the average RH dropped from 65.04% at an AQI < 75 to 42.17% at an AQI > 75), which weakened the aerosol water uptake. Figure 7b shows the variation in the mass share of the ALWC in the total PM_2.5_ under different AQI conditions, and it can be seen that the average mass contribution of the ALWC in PM_2.5_ was 13.27~15.13% at an AQI < 75, but decreased to 0.18~3.27% at an AQI > 75. Particularly, the ALWC only contributed ~0.18% of the PM_2.5_ mass in the polluted air (AQI > 100), indicating that the ALWC negligibly impacts the AQI, again due to the large AQI that was mainly caused by O_3_ not PM_2.5_ during the summer. Yet, we expect the ALWC to play an important role in air pollution events during the winter, when the AQI is mainly controlled by the PM pollution.

### 3.4. Relationship between ALWC and Visibility

As is well known, ALWC is an important factor influencing atmospheric visibility.

**Figure 8:**
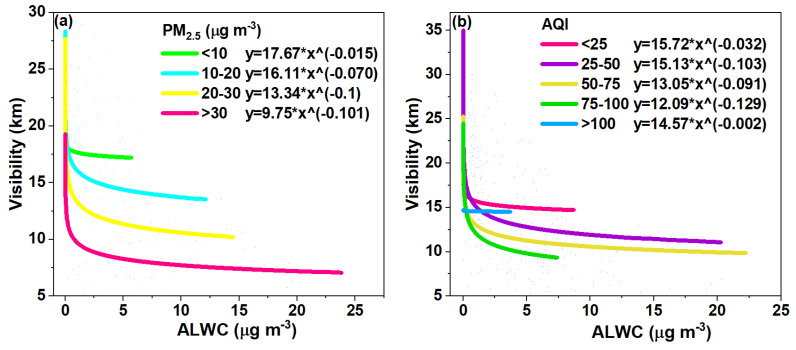
(**a**) Variation in visibility with ALWC for different PM_2.5_ concentrations; and (**b**) Variation in visibility with ALWC for different AQI values. The fitting equation used is y = a*x^b^.

Here, we further plotted the variations in visibility against the ALWC at different PM_2.5_ and AQI levels, respectively, in Figure 8a,b. It is very clear that the visibility degraded rapidly with the increase in PM_2.5_ levels and the exponential coefficients also increased with the PM_2.5_ levels, indicating the same amount of ALWC can lower the visibility even more significantly when the PM_2.5_ pollution is greater. As the ALWC increases, the aerosol size increases due to the uptake of water and can lead to a large increase in the aerosol extinction coefficient [61,62], which has been observed in various sites including Guangzhou, China and Delhi, India [63,64]. Overall, when the PM_2.5_ concentration was higher than 30 μg m^−3^, in most cases, the association with a very small amount of ALWC could lower the visibility to less than 10 km, i.e., hazy conditions; when the PM_2.5_ mass loading was between 20 and 30 μg m^−3^, the ALWC increased to ~15 μg m^−3^ and the visibility decreased to near 10 km, approaching hazy conditions. In other cases, the ALWC decreases the visibility but does not induce hazy conditions. This result is similar to that of a previous study, which shows that aerosols may have a great impact on visibility in summer when the PM_2.5_ concentration reaches a certain threshold [65].

On the other hand, Figure 8b shows the variation in visibility against the ALWC at different AQI levels. The visibility shows an even faster decrease with the increase in AQI levels, as can be roughly determined from the exponential coefficients in Figure 8b. The visibility can decrease to <10 km even at an AQI of 75–100, which means that the air quality is categorized as good (“clean air”), but when the ALWC is taken into consideration, the air can become hazy (“polluted air”) in many cases, highlighting the importance of the ALWC. This finding has important implications, as the inclusion of ALWC can affect air quality classifications, which should be taken into account in public health early warning and protection systems. Another interesting result is that the visibility sometimes was not less than 10 km when the AQI was larger than 100. This result was due to the reason explained earlier: large AQI values in the summer are mainly attributed to large O_3_ not PM_2.5_ levels; in addition, only a small portion of the data fell into the AQI > 100 range, making the fitting less robust compared to that of other cases.

### 3.5. Changes in ALWC in Different Air Parcels

**Figure 9:**
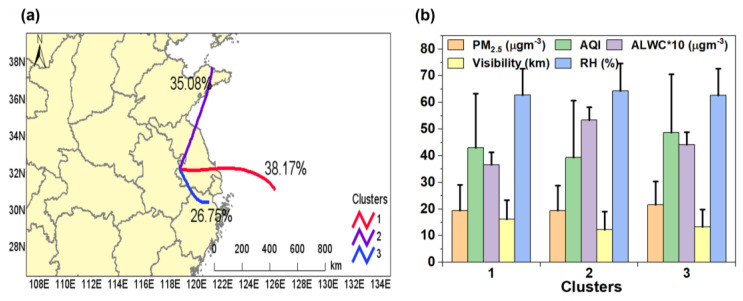
(**a**) Clustering results of the 36 h backward trajectories of air parcels arrived at the sampling site during the measurement period; (**b**) Average values of PM_2.5_, AQI, ALWC, visibility, and RH of the three clusters.

We explored the dependence of the ALWC on different air parcels, as PM_2.5_ with different origins differs in chemical compositions, thus affecting the ALWC [66,67,68]. The 36 h backward trajectories (initialized at a height of 500 m) were calculated by the MeteoInfo software (version 3.6) driven by the Global Data Assimilation System (GDAS) 1° × 1° reanalysis product [69]. The trajectories were grouped into three clusters: Cluster 1 (C1, 38.17% of the total) originated from the eastern sea; cluster 2 (C2, 35.08% of the total) began from the Shandong peninsula but intercepted sea air; and cluster 3 (C3, 26.75% of total) was the shortest, starting from Northern Zhejiang province. The mean values of the PM_2.5_, ALWC, AQI, and visibility of the three clusters are shown in Figure 9b. C2 had the highest ALWC (5.34 μg m^−3^), yet its visibility, PM_2.5_, and AQI were the lowest, mainly because C2 had the highest RH (64.36%) among the three clusters. On the other hand, the ALWC of C3 (4.42 μg m^−3^) was higher than that of C1 (3.66 μg m^−3^), mainly owing to the higher PM_2.5_ level of C3 (21.62 μg m^−3^) than C1 (19.48 μg m^−3^), as their RH values were very close (62.68% vs. 62.77%). Both the PM_2.5_ and AQI were the highest in C3, as it originated from an inland area and did not include clean sea air.

## 4. Conclusions

In this work, we first improved on our previous simple and low-cost method for the direct measurement of ALWC by replacing the highly expensive HR-AMS with a routine PM_2.5_ mass monitor (5030i) and with two turbidimeters (PDRs) in parallel. The improved suite of instruments was successfully applied to the ALWC measurement of PM_2.5_ in Nanjing for the summer of 2023. The average PM_2.5_ concentration was determined to be 19.50 μg m^−3^ and the ALWC was 3.85 μg m^−3^; therefore, the ALWC was ~16.5% of the total PM_2.5_ mass. The mass fraction of the ALWC also increased with the increase in the PM_2.5_ concentration, partially due to the increased mass contribution of hygroscopic secondary species. The ALWC positively correlated with the RH and PM_2.5_ concentrations, but negatively with the wind speed and temperature as well as the O_3_ concentration; correspondingly, it did not always increase with the air quality index (AQI) either, due to the fact that large AQI values in summer are often governed by O_3_ not PM_2.5_ levels and differ from those in winter.

Furthermore, we found that the ALWC also had a great impact on the visibility, which decreased with the ALWC rapidly at all PM_2.5_ levels. The decrease in the visibility at higher PM_2.5_ levels, however, appeared to be even quicker, dropping to <10 km when the PM_2.5_ was ~30 μg m^−3^ or the AQI was between 75 and 100. This result suggests that with the consideration of the ALWC, some days with “good” air quality (classified by the CAAQS PM_2.5_ standard or the AQI categories) might actually be “lightly polluted”. We also found that the air mass originating from Northeast China (the Shandong peninsula) had the lowest PM_2.5_ mass concentration yet the highest ALWC value mainly due to having the highest RH. In general, our findings presented here advance our understanding of aerosol chemistry and toxicology and may be valuable in future studies on air quality control in densely populated cities.

## Figures and Tables

**Table 1:** Representative ALWC levels in the YRD region, China.

Site	Sampling Period	Method	PM2.5	ALWC	RH (%)	Reference
			μg m^−3^			
Shanghai	23 October 2018 to 5 August 2019	ISORROPIA–II	38.60	14.80	70.00	[46]
Huangshan	14 September to 26 October 2012	AIM-II	21.28	9.98	64.50	[47]
Jiaxing	1 January to 31 December 2021	ISORROPIA–II	24.45	46.65	78.38	[48]
Nanjing	18 July to 26 August 2021	E-AIM	18.88 (PM_1_)	5.33	69.05	[20]

## Data Availability

The data presented in this article are available on request from the corresponding author.

## References

[1] Zhou Y., Zhang H., Parikh H.M., Chen E.H., Rattanavaraha W., Rosen E.P., Wang W., Kamens R.M. (2011). Secondary Organic Aerosol Formation from Xylenes and Mixtures of Toluene and Xylenes in an Atmospheric Urban Hydrocarbon Mixture: Water and Particle Seed Effects (II). Atmos. Environ..

[2] Kitamori Y., Mochida M., Kawamura K. (2009). Assessment of the Aerosol Water Content in Urban Atmospheric Particles by the Hygroscopic Growth Measurements in Sapporo, Japan. Atmos. Environ..

[3] Kreidenweis S.M., Petters M.D., DeMott P.J. (2008). Single-Parameter Estimates of Aerosol Water Content. Environ. Res. Lett..

[4] Nguyen T.K.V., Zhang Q., Jimenez J.L., Pike M., Carlton A.G. (2016). Liquid Water: Ubiquitous Contributor to Aerosol Mass. Environ. Sci. Technol. Lett..

[5] Dougle P.G., Vlasenko A.L., Veefkind J.P., Ten Brink H.M. (1996). Humidity Dependence of the Light Scattering by Mixtures of Ammonium Nitrate, Ammonium Sulfate and Soot. J. Aerosol Sci..

[6] Kuang Y., Zhao C.S., Tao J.C., Bian Y.X., Ma N. (2016). Impact of Aerosol Hygroscopic Growth on the Direct Aerosol Radiative Effect in Summer on North China Plain. Atmos. Environ..

[7] Liao H. (2005). Global Impacts of Gas-Phase Chemistry-Aerosol Interactions on Direct Radiative Forcing by Anthropogenic Aerosols and Ozone. J. Geophys. Res..

[8] Steinfeld J.I. (1998). Atmospheric Chemistry and Physics: From Air Pollution to Climate Change. Environ. Sci. Policy Sustain. Dev..

[9] Tao J.C., Zhao C.S., Ma N., Liu P.F. (2014). The Impact of Aerosol Hygroscopic Growth on the Single-Scattering Albedo and Its Application on the NO_2_ Photolysis Rate Coefficient. Atmos. Chem. Phys..

[10] Martin S.T. (2000). Phase Transitions of Aqueous Atmospheric Particles. Chem. Rev..

[11] Arellanes C., Paulson S.E., Fine P.M., Sioutas C. (2006). Exceeding of Henry’s Law by Hydrogen Peroxide Associated with Urban Aerosols. Environ. Sci. Technol..

[12] Cheng Y., Zheng G., Wei C., Mu Q., Zheng B., Wang Z., Gao M., Zhang Q., He K., Carmichael G. (2016). Reactive Nitrogen Chemistry in Aerosol Water as a Source of Sulfate during Haze Events in China. Sci. Adv..

[13] Wang G., Zhang R., Gomez M.E., Yang L., Levy Zamora M., Hu M., Lin Y., Peng J., Guo S., Meng J. (2016). Persistent Sulfate Formation from London Fog to Chinese Haze. Proc. Natl. Acad. Sci. USA.

[14] Jiang S.Y.N., Yang F., Chan K.L., Ning Z. (2014). Water Solubility of Metals in Coarse PM and PM 2.5 in Typical Urban Environment in Hong Kong. Atmos. Pollut. Res..

[15] Oakes M., Ingall E.D., Lai B., Shafer M.M., Hays M.D., Liu Z.G., Russell A.G., Weber R.J. (2012). Iron Solubility Related to Particle Sulfur Content in Source Emission and Ambient Fine Particles. Environ. Sci. Technol..

[16] Shiraiwa M., Ueda K., Pozzer A., Lammel G., Kampf C.J., Fushimi A., Enami S., Arangio A.M., Fröhlich-Nowoisky J., Fujitani Y. (2017). Aerosol Health Effects from Molecular to Global Scales. Environ. Sci. Technol..

[17] Liu M., Wang W., Li J., Wang T., Xu Z., Song Y., Zhang W., Zhou L., Lian C., Yang J. (2022). High Fraction of Soluble Trace Metals in Fine Particles under Heavy Haze in Central China. Sci. Total Environ..

[18] Wexler A.S. (2002). Atmospheric Aerosol Models for Systems Including the Ions H^+^, NH_4_^+^, Na^+^, SO_4_^2−^, NO_3_^−^, Cl^−^, Br^−^, and H_2_O. J. Geophys. Res..

[19] Fountoukis C., Nenes A. (2007). ISORROPIA II: A Computationally Efficient Thermodynamic Equilibrium Model for K^+^–Ca^2+^–Mg^2+^–NH^4+^ –Na^+^–SO_4_^2−^–NO_3_^−^–Cl^−^–H_2_O Aerosols. Atmos. Chem. Phys..

[20] Xian J., Cui S., Chen X., Wang J., Xiong Y., Gu C., Wang Y., Zhang Y., Li H., Wang J. (2023). Online Chemical Characterization of Atmospheric Fine Secondary Aerosols and Organic Nitrates in Summer Nanjing, China. Atmos. Res..

[21] Zhang Y., Tang L., Croteau P.L., Favez O., Sun Y., Canagaratna M.R., Wang Z., Couvidat F., Albinet A., Zhang H. (2017). Field Characterization of the PM_2.5_Aerosol Chemical Speciation Monitor: Insights into the Composition, Sources, and Processes of Fine Particles in Eastern China. Atmos. Chem. Phys..

[22] Wu Z.J., Zheng J., Shang D.J., Du Z.F., Wu Y.S., Zeng L.M., Wiedensohler A., Hu M. (2016). Particle Hygroscopicity and Its Link to Chemical Composition in the Urban Atmosphere of Beijing, China, during Summertime. Atmos. Chem. Phys..

[23] Bian Y.X., Zhao C.S., Ma N., Chen J., Xu W.Y. (2014). A Study of Aerosol Liquid Water Content Based on Hygroscopicity Measurements at High Relative Humidity in the North China Plain. Atmos. Chem. Phys..

[24] Jin X., Wang Y., Li Z., Zhang F., Xu W., Sun Y., Fan X., Chen G., Wu H., Ren J. (2020). Significant Contribution of Organics to Aerosol Liquid Water Content in Winter in Beijing, China. Atmos. Chem. Phys..

[25] Tan H., Cai M., Fan Q., Liu L., Li F., Chan P.W., Deng X., Wu D. (2017). An Analysis of Aerosol Liquid Water Content and Related Impact Factors in Pearl River Delta. Sci. Total Environ..

[26] Dai H., Zhang J., Gui H., Shen L., Wei X., Xie Z., Chen S., Wu Z., Chen D.-R., Liu J. (2022). Characteristics of Aerosol Size Distribution and Liquid Water Content under Ambient RH Conditions in Beijing. Atmos. Environ..

[27] Engelhart G.J., Hildebrandt L., Kostenidou E., Mihalopoulos N., Donahue N.M., Pandis S.N. (2011). Water Content of Aged Aerosol. Atmos. Chem. Phys..

[28] Jia L., Xu Y. (2018). Different Roles of Water in Secondary Organic Aerosol Formation from Toluene and Isoprene. Atmos. Chem. Phys..

[29] Davis E.J., Ray A.K. (1977). Determination of Diffusion Coefficients by Submicron Droplet Evaporation. J. Chem. Phys..

[30] Roberts G.C., Nenes A. (2015). A Continuous-Flow Streamwise Thermal-Gradient CCN Chamber for Atmospheric Measurements. Aerosol Sci. Technol..

[31] Rose D., Gunthe S.S., Mikhailov E., Frank G.P., Dusek U., Andreae M.O., Poschl U. (2008). Calibration and Measurement Uncertainties of a Continuous-FLow Cloud Condensation Nuclei Counter (DMT-CCNC): CCN Activation of Ammonium Sulfate and Sodium Chloride Aerosol Particles in Theory and Experiment. Atmos. Chem. Phys..

[32] Tan W., Yu Y., Li C., Li J., Kang L., Dong H., Zeng L., Zhu T. (2020). Profiling Aerosol Liquid Water Content Using a Polarization Lidar. Environ. Sci. Technol..

[33] Kuang Y., Zhao C.S., Zhao G., Tao J.C., Xu W., Ma N., Bian Y.X. (2018). A Novel Method for Calculating Ambient Aerosol Liquid Water Content Based on Measurements of a Humidified Nephelometer System. Atmos. Meas. Tech..

[34] Zhang J., Lance S., Wang X., Wang J., Schwab J.J. (2020). Estimation of Aerosol Liquid Water from Optical Scattering Instruments Using Ambient and Dried Sample Streams. Atmos. Environ..

[35] Wang J., Yue H., Cui S., Zhang Y., Li H., Wang J., Ge X. (2022). Chemical Characteristics and Source-Specific Health Risks of the Volatile Organic Compounds in Urban Nanjing, China. Toxics.

[36] Nie D., Chen M., Wu Y., Ge X., Hu J., Zhang K., Ge P. (2018). Characterization of Fine Particulate Matter and Associated Health Burden in Nanjing. Int. J. Environ. Res. Public Health.

[37] Feng L., Zhou H., Chen M., Ge X., Wu Y. (2023). Computational and Experimental Assessment of Health Risks of Fine Particulate Matter in Nanjing and Yangzhou, China. Environ. Sci. Pollut. Res..

[38] Zhang J., Ninneman M., Joseph E., Schwab M.J., Shrestha B., Schwab J.J. (2020). Mobile Laboratory Measurements of High Surface Ozone Levels and Spatial Heterogeneity During LISTOS 2018: Evidence for Sea Breeze Influence. J. Geophys. Res. Atmos..

[39] Zhang J., Marto J.P., Schwab J.J. (2018). Exploring the Applicability and Limitations of Selected Optical Scattering Instruments for PM Mass Measurement. Atmos. Meas. Tech..

[40] DeCarlo P.F., Kimmel J.R., Trimborn A., Northway M.J., Jayne J.T., Aiken A.C., Gonin M., Fuhrer K., Horvath T., Docherty K.S. (2006). Field-Deployable, High-Resolution, Time-of-Flight Aerosol Mass Spectrometer. Anal. Chem..

[41] Zhang Y., Wang J., Cui S., Huang D.D., Ge X. (2020). Aerosol Measurements by Soot Particle Aerosol Mass Spectrometer: A Review. Curr. Pollut. Rep..

[42] Canagaratna M.R., Jayne J.T., Jimenez J.L., Allan J.D., Alfarra M.R., Zhang Q., Onasch T.B., Drewnick F., Coe H., Middlebrook A. (2007). Chemical and Microphysical Characterization of Ambient Aerosols with the Aerodyne Aerosol Mass Spectrometer. Mass Spectrom. Rev..

[43] Joo T., Chen Y., Xu W., Croteau P., Canagaratna M.R., Gao D., Guo H., Saavedra G., Kim S.S., Sun Y. (2021). Evaluation of a New Aerosol Chemical Speciation Monitor (ACSM) System at an Urban Site in Atlanta, GA: The Use of Capture Vaporizer and PM_2.5_Inlet. ACS Earth Space Chem..

[44] Li S., Chen C., Yang G., Fang J., Sun Y., Tang L., Wang H., Xiang W., Zhang H., Croteau P.L. (2022). Sources and Processes of Organic Aerosol in Non-Refractory PM1 and PM_2.5_ during Foggy and Haze Episodes in an Urban Environment of the Yangtze River Delta, China. Environ. Res..

[45] Crutzen P. (2001). Human Impacts on Atmospheric Chemistry. Annu. Rev. Earth Planet. Sci..

[46] Duan X., Yan Y., Peng L., Xie K., Hu D., Li R., Wang C. (2021). Role of Ammonia in Secondary Inorganic Aerosols Formation at an Ammonia-Rich City in Winter in North China: A Comparative Study among Industry, Urban, and Rural Sites. Environ. Pollut..

[47] Li L., Yin Y., Kong S., Wen B., Chen K., Yuan L., Li Q. (2014). Altitudinal Effect to the Size Distribution of Water Soluble Inorganic Ions in PM at Huangshan, China. Atmos. Environ..

[48] Shi R., Zhang F., Shen Y., Shen J., Xu B., Kuang B., Xu Z., Jin L., Tang Q., Tian X. (2024). Aerosol Liquid Water in PM_2.5_ and Its Roles in Secondary Aerosol Formation at a Regional Site of Yangtze River Delta. J. Environ. Sci..

[49] Wang J., Ge X., Chen Y., Shen Y., Zhang Q., Sun Y., Xu J., Ge S., Yu H., Chen M. (2016). Highly Time-Resolved Urban Aerosol Characteristics during Springtime inYangtze River Delta, China: Insights from Soot Particle Aerosol Massspectrometry. Atmos. Chem. Phys..

[50] Wang J., Ge X., Sonya C., Ye J., Lei Y., Chen M., Zhang Q. (2022). Influence of Regional Emission Controls on the Chemical Composition, Sources, and Size Distributions of Submicron Aerosols: Insights from the 2014 Nanjing Youth Olympic Games. Sci. Total Environ..

[51] Li X., Song S., Zhou W., Hao J., Worsnop D.R., Jiang J. (2019). Interactions between Aerosol Organic Components and Liquid Water Content during Haze Episodes in Beijing. Atmos. Chem. Phys..

[52] Wu Z., Wang Y., Tan T., Zhu Y., Li M., Shang D., Wang H., Lu K., Guo S., Zeng L. (2018). Aerosol Liquid Water Driven by Anthropogenic Inorganic Salts: Implying Its Key Role in Haze Formation over the North China Plain. Environ. Sci. Technol. Lett..

[53] He J., Gong S., Yu Y., Yu L., Wu L., Mao H., Song C., Zhao S., Liu H., Li X. (2017). Air Pollution Characteristics and Their Relation to Meteorological Conditions during 2014–2015 in Major Chinese Cities. Environ. Pollut..

[54] Zhang Y.-L., Cao F. (2015). Fine Particulate Matter (PM_2.5_) in China at a City Level. Sci. Rep..

[55] Shen F., Ge X., Hu J., Nie D., Tian L., Chen M. (2017). Air Pollution Characteristics and Health Risks in Henan Province, China. Environ. Res..

[56] Sun Y., Chen C., Zhang Y., Xu W., Zhou L., Cheng X., Zheng H., Ji D., Li J., Tang X. (2016). Rapid Formation and Evolution of an Extreme Haze Episode in Northern China during Winter 2015. Sci. Rep..

[57] Wang Y., Zhang F., Li Z., Tan H., Xu H., Ren J., Zhao J., Du W., Sun Y. (2017). Enhanced Hydrophobicity and Volatility of Submicron Aerosols under Severe Emission Control Conditions in Beijing. Atmos. Chem. Phys..

[58] Monteiro A., Vieira M., Gama C., Miranda A.I. (2017). Towards an Improved Air Quality Index. Air Qual. Atmos. Health.

[59] Shen F., Zhang L., Jiang L., Tang M., Gai X., Chen M., Ge X. (2020). Temporal Variations of Six Ambient Criteria Air Pollutants from 2015 to 2018, Their Spatial Distributions, Health Risks and Relationships with Socioeconomic Factors during 2018 in China. Environ. Int..

[60] Lei R., Nie D., Zhang S., Yu W., Ge X., Song N. (2022). Spatial and Temporal Characteristics of Air Pollutants and Their Health Effects in China during 2019–2020. J. Environ. Manag..

[61] Chen J., Zhao C.S., Ma N., Yan P. (2014). Aerosol Hygroscopicity Parameter Derived from the Light Scattering Enhancement Factor Measurements in the North China Plain. Atmos. Chem. Phys..

[62] Liu X., Gu J., Li Y., Cheng Y., Qu Y., Han T., Wang J., Tian H., Chen J., Zhang Y. (2013). Increase of Aerosol Scattering by Hygroscopic Growth: Observation, Modeling, and Implications on Visibility. Atmos. Res..

[63] Ali U., Faisal M., Ganguly D., Kumar M., Singh V. (2023). Analysis of Aerosol Liquid Water Content and Its Role in Visibility Reduction in Delhi. Sci. Total Environ..

[64] Jin X., Li Z., Wu T., Wang Y., Cheng Y., Su T., Wei J., Ren R., Wu H., Li S. (2022). The Different Sensitivities of Aerosol Optical Properties to Particle Concentration, Humidity, and Hygroscopicity between the Surface Level and the Upper Boundary Layer in Guangzhou, China. Sci. Total Environ..

[65] Zhao P., Zhang X., Xu X., Zhao X. (2011). Long-Term Visibility Trends and Characteristics in the Region of Beijing, Tianjin, and Hebei, China. Atmos. Res..

[66] Asmi E., Frey A., Virkkula A., Ehn M., Manninen H.E., Timonen H., Tolonen-Kivimäki O., Aurela M., Hillamo R., Kulmala M. (2010). Hygroscopicity and Chemical Composition of Antarctic Sub-Micrometre Aerosol Particles and Observations of New Particle Formation. Atmos. Chem. Phys..

[67] Cai M., Tan H., Chan C.K., Mochida M., Hatakeyama S., Kondo Y., Schurman M.I., Xu H., Li F., Shimada K. (2017). Comparison of Aerosol Hygroscopcity, Volatility, and Chemical Composition between a Suburban Site in the Pearl River Delta Region and a Marine Site in Okinawa. Aerosol. Air Qual. Res..

[68] Hong J., Kim J., Nieminen T., Duplissy J., Ehn M., Äijälä M., Hao L.Q., Nie W., Sarnela N., Prisle N.L. (2015). Relating the Hygroscopic Properties of Submicron Aerosol to Both Gas- and Particle-Phase Chemical Composition in a Boreal Forest Environment. Atmos. Chem. Phys..

[69] Petit J.-E., Favez O., Albinet A., Canonaco F. (2017). A User-Friendly Tool for Comprehensive Evaluation of the Geographical Origins of Atmospheric Pollution: Wind and Trajectory Analyses. Environ. Model. Softw..

